# A named GP increases self-reported access to health care services

**DOI:** 10.1186/s12913-022-08660-5

**Published:** 2022-10-19

**Authors:** Emmi Lautamatti, Kari Mattila, Sakari Suominen, Lauri Sillanmäki, Markku Sumanen

**Affiliations:** 1 Faculty of Medicine and Health Technology, Tampere University, Tampere, Finland; 2 Centre for General Practice, Pirkanmaa Hospital District, Tampere, Finland; 3 School of Health Sciences, University of Skövde, Skövde, Sweden; 4 Department of Public Health and Clinical Research Centre, University of Turku, Turku University Hospital, Turku, Finland; 5 Turku University Hospital and University of Turku, Turku, Finland; 6 Department of Public Health, University of Helsinki, Helsinki, Finland

**Keywords:** Continuity of care, Accessibility, Health care, Primary care, Hospital care, Named GP, Use of health care services

## Abstract

**Background:**

Continuity of care strengthens health promotion and decreases mortality, although the mechanisms of these effects are still unclear. In recent decades, continuity of care and accessibility of health care services have both decreased in Finland.

**Objectives:**

The aim of the study was to investigate whether a named and assigned GP representing continuity of care is associated with the use of primary and hospital health care services and to create knowledge on the state of continuity of care in a changing health care system in Finland.

**Methods:**

The data are part of the Health and Social Support (HeSSup) mail survey based on a random Finnish working age population sample of 64,797 individuals drawn in 1998 and follow-up surveys in 2003 and 2012. The response rate in 1998 was 40% (*n* = 25,898). Continuity of care was derived from the 2003 and 2012 data sets, other variables from the 2012 survey (*n* = 11,924). The principal outcome variables were primary health care and hospital service use reported by participants. The association of the explanatory variables (gender, age, education, reported chronic diseases, health status, smoking, obesity, NYHA class of any functional limitation, depressive mood and continuity of care) with the outcome variables was analysed by binomial logistic regression analysis.

**Results:**

A named and assigned GP was independently and significantly associated with more frequent use of primary and hospital care in the adjusted logistic regression analysis (ORs 1.53 (95% CI 1.35–1.72) and 1.19 (95% CI 1.08–1.32), *p* < 0.001).

**Conclusion:**

A named GPs is associated with an increased use of primary care and hospital services. A named GP assures access to health care services especially to the chronically ill population. The results depict the state of continuity of care in Finland. All benefits of continuity of care are not enabled although it still assures treatment of population in the most vulnerable position.

## Introduction

Optimally functioning primary health care constitutes the principal basis of a high-quality health care system [1]. This implies equal service accessibility in a correct temporal order. The structure, process and outcome of services should all be monitored and analysed in an organisation producing high-quality health care services [2]. Continuity of care has been described as an important part of well-organised primary health care [1, 3, 4].

Continuity of care is cost-effective and should be taken into consideration in the production of health care services [5]. It improves quality of life and general life satisfaction, and it decreases all-cause mortality and the use of hospital services [6–8]. Considering hospital use, continuity of primary care shows an association with shorter hospitalisation periods and a decrease in unexpected hospitalisations [9, 10]. Continuity of care has been shown to be associated with medication adherence and the increased use of preventive medical care [11, 12]. Continuity of care has multiple dimensions (informational, managemental and relational), of which relational continuity has been the most studied one with Continuity of Care Index (COCI) and Usual Provider Index (UPI) as widely used indicators [3, 7]. The majority of benefits of continuity of care have been found while investigating relational continuity. In order to add knowledge of the mechanisms delivering the benefits relational continuity was also used in the present study. Hence, while the benefits of continuity of care are undisputed, the mechanisms transferring the effect are still unclear. Hence, continuity of care calls for further analytic studies in versatile health care organisations [8].

In Finland, the production of public health care services is presently legislated as the responsibility of municipalities [13]. During the observation period there were 309–415 municipalities with varying population sizes. The population uses publicly financed services in health centres and hospitals. In health centres, the population is offered primary medical services and preventive health care in combination with rehabilitation services. The focus of the work at the health centres in Finland is on preventive care and the treatment of common long-term illnesses [14]. In hospital specialist consultations are offered as ambulatory and inpatient service. A patient needs a referral from primary health care to be entitled to enter a publicly funded hospital for a non-urgent matter. However, health care services are also provided by the private sector and referrals from the private sector are likewise accepted. When using the private sector, patients are entitled to a minor reimbursement provided by the publicly funded national Social Insurance Institution. The employed working-age population usually also has access to occupational health services, which are provided mainly by private producers financed by the employer.

Historically in Finland, the municipalities addressed a named and assigned GP for each citizen in primary care. A trial in 1980s demonstrated the benefits of the system [15]. A named GP established the interpersonal continuity of care [7]. After the economic depression in 1990s the lack of GPs accompanied with decreasing public funding forced municipalities to find alternative models of service production. A personal listing or team-based care became the new basis of primary care. The historical background clarifies the absence of national registries of continuity of care in Finland.

In recent decades in Finland, continuity of care as well as accessibility of primary care have deteriorated [16]. In the studies a named GP has been a valid proxy for continuity of care [15, 16]. The lack of GP and nurse resources in primary health care affects temporal accessibility and produces local inequity of access to health care services. The crisis of primary care has been acknowledged [17] and the present government is establishing a health care and social service reform aimed at improved accessibility and equity of services [18].

Besides continuity of care there are several other factors affecting population’s use of health services. The number of chronic diseases, health status, smoking, obesity and functional limitations are associated with higher risk of hospitalisation [19–21]. In order to investigate associations between these factors and at the same time to test the reliability of our data we included them as explanatory variables in the present study as independent covariates.

There were two principal aims in the study: the first one was to study the association of named GP as a factor of continuity of care with the use of health centre and hospital services among Finnish population. The second aim was to produce information on the state of continuity of care in Finland. An additional aim was to compare significant explanatory variables of the use of primary or specialised health care.

## Methods

**Fig. 1:**
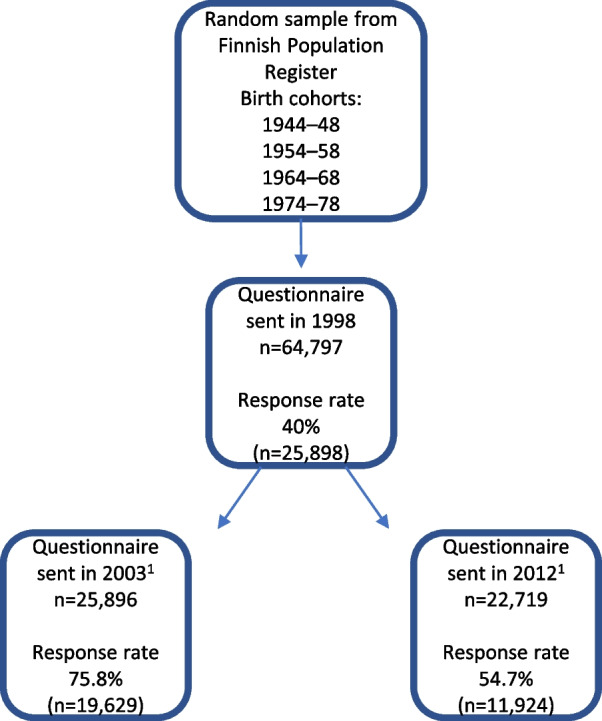
The procedure of forming the data for the study. 1) Participants who were deceased, had emigrated, or had declined delivery of their address from the Finnish Population register were excluded

The data are part of the Health and Social Support (HeSSup) study. In 1998, a questionnaire was sent to four birth cohorts (1944–1948, 1954–1958, 1964–1968 and 1974–1978) comprising 64,797 randomly selected individuals drawn from the Finnish Population Registry. The survey was repeated in 2003 and 2012 (response rates of 40.0%, 75.8% and 54.7%, respectively, calculated from the respondents of the preceding survey). Participants who had deceased, emigrated**,** or declined delivery of their address from the Finnish Population Register were excluded (Fig. 1). The data can be considered representative of the corresponding Finnish population [22, 23], as a careful non-response analysis in 1998 indicated that there were no factors disputing the comparability of respondents and non-respondents [22]. Most of the study variables were constructed based on cross-sectional data from the 2012 survey. The main interest was to study the self-reported use of health care services. A named GP was calculated from the data of the 2003 and 2012 surveys, respectively.

### Outcome variables

There were two outcome variables: use of health centre services and use of hospital services.

Use of health centre services was determined by a question on how many times the participant had visited a doctor at a health centre during the last twelve months. The alternatives were 0, 1, 2‒4 and 5 or more. The response was dichotomised into “0‒1” as zero to one visit to the services and “ > 1” as two or more visits to the health centre’s GP. The cut-off was based on the argumentation that one visit could be a random one.

Use of hospital services was calculated from three different survey items. The respondent was asked: “How many times during the last twelve months have you visited a doctor at a hospital polyclinic?”. The alternatives were 0, 1, 2‒4 and 5 or more. Dichotomisation was made to “No” indicating no use of the ambulatory service and “Yes” indicating one or more visits. The respondent was also asked: “During the last twelve months, has a doctor ordered or recommended hospital examinations or care for you?” and “During the last twelve months, has a doctor ordered or recommended examinations by a specialist for you?”. The alternatives to both questions were “No” and “Yes”. If the answer to any of the three questions was “Yes”, the participant was classified as having used hospital services. Ambulatory services were used with examinations by a specialist. Hospital examinations or hospital care demanded inpatient treatment. The cut-off points were chosen to describe any use of hospital services, urgent or planned, considering the special features of Finnish health care. Hence, the outcome variable of hospital days describes the general use of hospital services.

### Explanatory variables

Explanatory variables comprise four social background variables (gender, age and education), six health or health behaviour variables (chronic diseases, reported health status, smoking, obesity (BMI), functional limitations (NYHA) and depressive mood (BDI)) and one regional service characteristics (named GP). Besides age, all explanatory variables were dichotomised.

### Social background

Gender (male, female) comprised initially two categories. Age was categorised into four classes according to the birth cohorts (1944–1948, 1954–1958, 1964–1968 and 1974–1978). Participants with a degree from a college, university or polytechnic were considered as having higher education, whereas the rest were considered to have a low level of education.

### Health and health behaviour

Chronic diseases were inquired by “Has a physician ever said that you have or have had…” including 32 individual disease alternatives. The subject was able to select from the absence or existence of a disease mentioned by the physician. Boxes left blank were categorised as “No”. In total, 26 of the 32 diseases reported here were categorised as chronic. The National Institute for Health and Care Excellence (NICE) guidelines were used in the categorisations [24]. Participants in the “Chronic disease” group reported one or more chronic diseases. The rest formed the “No chronic disease” group.

Health status was inquired in the survey with a five-point Likert scale. The participants’ conception of their health as good or fairly good was categorised as good. Other assessments were considered poor based on the argumentation that the respondent did not at least consider her/his health as good. Smoking was dichotomised as “Yes” when the respondent reported being a smoker at the time of the survey and otherwise “No”. Body Mass Index (BMI) was cut into two categories with a cut-point of 25 kg/m^2^. The New York Heart Association (NYHA) classification describes the functional limitations of the respondent. All-cause exertional shortness of breath was inquired in the survey and scored according to the NYHA classification [25]. The NYHA scores were categorised into 0–1 and 2–4, with 0 meaning no symptoms during exercise or physical activity. Beck’s Depression Inventory (BDI) was used to assess the participants’ depressive mood [26]. Values < 19 were considered a normal or mildly depressive mood, while participants with values ≥ 19 were considered to have moderate or difficult depression (Table 1).

Participants reporting a named GP was determined in 2003 and 2012 with a single question: “Do you have an assigned and named GP at your local health centre?” The options “Yes” and “No” were available to indicate the existence or lack of named GP. Participants who answered “Yes” in both years were categorised as “Yes”. Others were categorised as “No”. The two surveys were combined to gather a wider perspective of continuity of care. The analyses were also carried out with only the study population from the 2012 survey with principally unchanged results (no data provided).

Binary logistic regression analysis was chosen as study method, since the outcome variables were binary and the observations were independent. There was no statistically significant multicollinearity among the explanatory variables, and the sample size was large (no data provided). The explanatory variables were dichotomised to highlight the most important results.

Only variables with a statistically significant association (p ≤ 0.05) with the dependent factor in the univariate logistic regression analysis were included in the multivariate analysis. Health status and chronic diseases were analysed as adjusting covariates both in the unadjusted and adjusted models.

The interaction effect of a named GP and primary care use in relation to hospital service use was studied. Likewise, the interaction effect of a named GP and hospital use in relation to the use of primary care was analysed too.

Characteristics were analysed by cross-tabulation and statistical analysis by binomial logistic regression analysis using IBM SPSS Statistics, version 27 and SAS software 9.4 TS1M5 [27, 28].

## Results

### Health centre services

More than one visit to the health centre during the last twelve months was reported by 25% (*n* = 2,886) of participants. The study population with a named GP reported at least one visit significantly more often (30.5% vs 20.0%) compared to the population with no named and assigned GP (*p* < 0.001) (Table 1).

**Table 1:** Characteristics and numbers of reported health centre visits (> 1) and hospital visits (yes) during the last twelve months. Data from the HeSSup questionnaire in 2012 (*n* = 11,924)

	Health centre visits				Hospital visits			
Characteristics	Total	> 1	Difference		Total	Yes	Difference	
	*n*	*n* (%)	% units	*p*	*n*	*n* (%)	% units	*p*
SOCIAL FACTORS								
Gender			-3.4	** < 0.001**			-3.7	** < 0.001**
Male	4269	974 (22.8)			3969	1543 (38.9)		
Female	7292	1912 (26.2)			6735	2870 (42.6)		
Birth cohort				** < 0.001**				** < 0.001**
1974–78	2440	495 (20.3)			2330	847 (36.4)		
1964–68	2391	461 (19.3)			2288	872 (38.1)		
1954–58	3058	637 (20.8)			2865	1231 (43.0)		
1944–48	3672	1293 (35.2)			3221	1463 (45.4)		
Native language			-1.5	0.271			2.5	0.127
Finnish	10,397	2580 (24.8)			9636	3996 (41.5)		
Swedish	1164	306 (26.3)			1068	417 (39.0)		
Education			10.5	** < 0.001**			3.0	**0.002**
Lower	7704	2181 (28.3)			7041	2973 (42.2)		
Higher	3784	674 (17.8)			3612	1415 (39.2)		
HEALTH STATUS								
Chronic disease			-18.6	** < 0.001**			-22.6	** < 0.001**
No	3684	453 (12.3)			3531	921 (26.1)		
Yes	7877	2433 (30.9)			7173	3492 (48.7)		
Health status			-32.2	** < 0.001**			-33.4	** < 0.001**
Good	10,858	2513 (23.1)			10,121	4001 (39.5)		
Poor	645	357 (55.4)			539	393 (72.9)		
Smoking			-4.0	**0.001**			-0.7	0.598
No	9149	2239 (24.5)			8511	3497 (41.1)		
Yes	1490	425 (28.5)			1367	572 (41.8)		
Obesity (BMI)			-7.4	** < 0.001**			-5.4	** < 0.001**
< 25 kg/m^2^	4984	1033 (20.7)			4646	1776 (38.2)		
≥ 25 kg/m^2^	6453	1814 (28.1)			5947	2591 (43.6)		
Functional limitations			-26.7	** < 0.001**			-21.6	** < 0.001**
NYHA 0–1	10,396	2320 (22.3)			9700	3806 (39.2)		
NYHA 2–4	1118	548 (49.0)			972	591 (60.8)		
Depressive mood (BDI)			-18.5	** < 0.001**			-15.8	** < 0.001**
< 19	10,926	2634 (24.1)			10,158	4116 (40.5)		
≥ 19	519	221 (42.6)			460	259 (56.3)		
REGIONAL SERVICE CHARACTERISTICS								
Named GP^a^			-10.5	** < 0.001**			-5.0	** < 0.001**
No	3192	637 (20.0)			2992	1147 (38.3)		
Yes	4007	1222 (30.5)			3698	1601 (43.3)		

^a^Measured longitudinally from the 2003 and 2012 questionnaires

In the unadjusted logistic regression analysis, the odds ratio for more than one health centre visit was 2.24 with a NYHA score of 2‒4 (95% CI 1.95–2.57). Among the 1944‒1948 birth cohort, the odds ratio was 1.58 (95% CI 1.39–1.79), while among participants with a named GP it was 1.65 (95% CI 1.47–1.84). In the adjusted model, a named GP was significantly associated with the use of health centre services more than once during the last twelve months (OR 1.53; 95% CI 1.35–1.72) A higher NYHA score showed the strongest associations, with an odds ratio of 2.51 for two or more visits (95% CI 2.10–3.00) (Table 2). Interaction between named GP and hospital service use was not statistically significant when predicting primary care use (data not shown).

**Table 2:** Binomial logistic regression on reported use of health centre services among participants of the 2012 HeSSup questionnaire. Determining the association between independent factors and more than one visit to a health centre during the last twelve months*

	Use of health centre services				
Characteristics	Unadjusted			Adjusted**	
	OR (95% Cl)	*p*		OR (95% Cl)	*p*
Social factors					
Gender		**0.001**			** < 0.001**
Male	1			1	
Female	**1.14 (1.04–1.25)**			**1.21 (1.06–1.37)**	
Birth cohort		** < 0.001**			** < 0.001**
1974–78	1			1	
1964–68	0.86 (0.74–0.99)	**0.04**		0.85 (0.70–1.03)	0.097
1954–58	0.80 (0.70–0.92)	**0.001**		0.84 (0.70–1.01)	0.07
1944–48	**1.58 (1.39–1.79)**	** < 0.001**		**1.64 (1.37–1.95)**	** < 0.001**
Education		** < 0.001**			** < 0.001**
Higher	1			1	
Lower	**1.58 (1.43–1.75)**			**1.44 (1.25–1.66)**	
Health status					
Smoking		**0.05**			0.180
No	1			1	
Yes	**1.14 (1.00–1.29)**			1.12 (0.95–1.32)	
Obesity (BMI)		** < 0.001**			** < 0.001**
< 25 kg/m^2^	1			1	
≥ 25 kg/m^2^	**1.34 (1.23–1.47)**			**1.28 (1.13–1.45)**	
NYHA classification		** < 0.001**			** < 0.001**
0–1	1			1	
2–4	**2.24 (1.95–2.57)**			**2.51 (2.10–3.00)**	
Depressive mood (BDI)		**0.001**			** < 0.001**
< 19	1			1	
≥ 19	**1.39 (1.14–1.69)**			**1.79 (1.39–2.31)**	
Regional service characteristics					
Named GP		** < 0.001**			** < 0.001**
No	1		1		
Yes	**1.65 (1.47–1.84)**		**1.53 (1.35–1.72)**		

^*^ Health status and chronic diseases analysed as adjusting covariates in the unadjusted and adjusted models

^**^ Adjusted analysis includes factors that showed a statistically significant association with health centre visits in the unadjusted analysis

### Hospital services

Use of hospital services was reported by 41% (*n* = 4413) of participants. These services were used by a 5.0 percentage greater share of the population with a named GP than those lacking a named and assigned GP (*p* < 0.001) (Table 1).

In the unadjusted logistic regression analysis, NYHA class 2‒4 significantly increased the odds of hospital use (OR 1.58; 95% CI 1.37–1.83) as well as a named GP (OR 1.14; 95% CI 1.03–1.26) and obesity (OR 1.13; 95% CI 1.04–1.22). In the adjusted analysis, a named GP was positively associated (OR 1.19; 95% CI 1.08–1.32), while NYHA held the strongest association with the probability of hospital use, with an odds ratio of 2.21 (95% CI 1.86–2.63) (Table 3). Interaction between a named GP and primary care use was not statistically significant when predicting hospital service use (data not shown).

**Table 3:** Binomial logistic regression on the reported use of hospital services among participants of the 2012 HeSSup questionnaire. Determining the association between independent factors and hospital visits during the last twelve months*

	Use of hospital services			
Characteristics	Unadjusted		Adjusted**	
	OR (95% Cl)	*p*	OR (95% Cl)	*p*
Social factors				
Gender		**0.04**		**0.03**
Male	1		1	
Female	1.09 (1.01–1.19)		1.12 (1.01–1.24)	
Birth cohort		0.476		
1974–78	1			
1964–68	1.00 (0.89–1.14)	0.956		
1954–58	1.07 (0.96–1.21)	0.238		
1944–48	1.07 (0.95–1.20)	0.260		
Education		0.815		
Higher	1			
Lower	0.99 (0.91–1.08)			
Health status				
Smoking		0.495		
No	1			
Yes	0.96(0.85–1.08)			
Obesity (BMI)		**0.004**		**0.04**
< 25 kg/m^2^	1		1	
≥ 25 kg/m^2^	**1.13 (1.04–1.22)**		**1.12 (1.00–1.23)**	
NYHA classification		** < 0.001**		** < 0.001**
0–1	1		1	
2–4	**1.58 (1.37–1.83)**		**2.21 (1.86–2.63)**	
Depressive mood (BDI)		0.122		
< 19	1			
≥ 19	1.17 (0.96–1.44)			
Regional service characteristics				
Named GP		**0.013**		** < 0.001**
No	1		1	
Yes	**1.14 (1.03–1.26)**		**1.19 (1.08–1.32)**	

^*^ Health status and chronic diseases analysed as adjusting covariates

^**^ Adjusted analysis includes factors that showed a statistically significant association with hospital visits in the unadjusted analysis

## Discussion

A named GP was associated with the increased self-reported use of health centre and all-cause hospital services, which creates premises to establish continuity of care. The study provides new knowledge on the state of continuity of care in Finland.

The named and assigned GP represents continuity of care in the Finnish context. Although we don´t have data on interpersonal continuity, a named GP increases population´s satisfaction with health care services. [26]. The repetitive manner of reporting a named GP indicates a possibility and the participants’ preference to achieve a long-lasting patient-doctor relationship. In Finland, after decades of decrease in accessibility and continuity of care, reported named GP still has an association with health services use. The named GP seems to assure the access to the health care services. The finding encourages to assess continuity of care also at the population level, which is notified in previous studies as well [8].

A population with a named GP and continuity of care uses primary care services more, which increases the use of preventive and life-protective services. Despite the health-promoting effect of continuity of care, the population naturally continues to fall ill and is thus, in further need of health care services [3]. Nevertheless, people with health care continuity seem to pay more attention to their health than the rest of the population [11, 12, 29]. In longer relationships, trust and loyalty create a more evident effect [30], and there is evidence of the dose-dependent nature of continuity of care [31]. Continuity of care improves life quality [6] and decreases mortality [8, 32], which are addressed by repeated visits to primary care services.

A named GP representing continuity of care is also associated with an increased use of hospital services. The result is contradictory to some earlier studies [4, 10, 33, 34], although studies with supporting results have been published as well [33]. The definition of continuity of care varied in the studies as well as study population, although emphasising the older population with chronic diseases. In our interpretation, one reason for the difference might also lie in the definitions of hospital care.

Hospital service use in this study comprised hospitalisation but also external specialist services, which usually in Finland are represented by specialist consultations. The responsibility for total care and managing common public health concerns lies with the primary care physician. Still, the study did not focus on the associations with emergency room use or preventable hospitalisations, nor on referral or treatment quality. Thus, we cannot make assumptions regarding the necessity of the referrals to hospital care. We adopted a wider view of health care service use to find out the state of continuity of care. A named GP assures access to hospital services. The cut-off point for hospital services was selected with consideration of the special nature of the Finnish health care system and in view of our pursuit for a comprehensive picture of the total use of the services.

There are limitations concerning the study setting. All the results reflect the participants’ perceptions and are thus not objective. The items determining the use of hospital services measured the medically validated need for these kinds of services and not the final use. Use of services is also defined by the respondents, which is vulnerable to reporting bias, since those with a worse health status could overestimate their use. The data are generalisable to the Finnish population, for which the response bias is not considered as a major limitation, although worth to be noticed. Information is always lost when variables are categorised. However, that way we were better able to study our main questions related to absence/existence of service use. Named GP as a factor of continuity of care was asked using a single question and reported by the participants. Though it is recommended that continuity of care is assessed in a patient-centred way [34], there are no data on the actually established continuity of care among the respondents. Baker et al. found good face validity for patient-reported continuity, which makes us confident of the relevance of the study findings [8].

Health-related factors and chronic conditions are strongly related to the use of health care services. Thereby, we used self-assessed health and chronic diseases as adjusting variables in the logistic regression analysis. Most participants with chronic conditions reported implemented continuity of care, and those using hospital services seemed to suffer from more complex concerns: chronic diseases in combination with poor health status. The population with chronic conditions benefits from continuity of care the most [3, 8], and those with continuity are more satisfied with health care services despite their impaired health and higher age [35]. The care of multi-morbid patients should be especially noted in primary care [36]. National and international guides for treating the multi-morbid population have been established for health care providers to give comprehensive care to this special patient group [24, 37].

Fewer than half of the participants with a chronic disease visited a health centre during the last twelve months, which raises concerns over service accessibility. The health status of the respondents was poor, which indicates that the less frequent use of health centre services does not reflect a situation with illnesses in good treatment balance. The rather large proportion of patients using hospital services also raises concerns over whether there is causality concerning the high use of secondary care services and accessibility problems in primary care. In Finland, accessibility to services has decreased in recent decades, and this is one of the greatest concerns of the health care organisations [16, 38]. Although our study setting does not allow interpretations on accessibility, strengthening primary care might potentially decrease the use of secondary services [4, 5, 29]. Moreover, according to a previous study, health care costs in the population were reduced by increasing availability in primary care [39].

In the study a named GP in health centre increases use of both health centre and hospital service. The named GP seems to assure access to hospital services when needed. Use of health care services is most common among the population with chronic diseases, which possibly contributes to patient safety. It is possible, that with higher rate of continuity some use of hospital services could be avoided. The reasons for hospital use are yet to be studied.

The benefits of continuity of care are undisputable. The mechanisms to mediate the benefits are thought to lie within the long-term doctor-patient relationships. In the study a named GP, which represents continuity of care in Finland, increases access to health care services. In the circumstances with chattered continuity, health care system is focusing on treatment of illnesses. All benefits of continuity of care are not enabled although it still seems to assure treatment of population in the most vulnerable position. The results encourage us to continue studying continuity of care in a variety of health care systems.

## Conclusions

A named GPs is associated with the increased use of primary care and hospital services and assures access to health care services especially to chronically ill population. The results picture the state of continuity of care in Finland. All benefits of continuity of care are not enabled although it still assures treatment of population in the most vulnerable position. On the cusp of beginning a health and social service reform in Finland, continuity of care should be acknowledged.

## Data Availability

All data analysed during this study are included in this published article. The HeSSup studies has the ownership of the data and publishing the data more precisely demands a consent from the third party.

## Abbreviations

GP: General Practitioner
COCI: Continuity of Care Index
UPI: Usual Provider Index
HeSSup: Health and Social Support study
NYHA: New York Heart Association
SPSS: IBM SPSS Statistics program
NICE: National Institute for Health and Care Excellence
BMI: Body Mass Index
BDI: Beck’s Depression Inventory
OR: Odds Ratio
CI: Confidence Interval
ER: Emergency Room/Unit
